# Exposure to TDCPP Appears Widespread

**DOI:** 10.1289/ehp.121-a150

**Published:** 2013-05-01

**Authors:** Kellyn S. Betts

**Affiliations:** Kellyn S. Betts writes about environmental contaminants, hazards, and technology for solving environmental problems for publications including *Environmental Science & Technology*.

At a time of growing debate over the safety and usefulness of flame retardants added to consumer products,^1^ new evidence is emerging about the breadth of human exposure to one of the most widely used of these compounds. Two new studies^2^^,^^3^ document the presence of the flame retardant tris(1,3-dichloro-2-propyl) phosphate (TDCPP) in dust from homes, offices, and automobiles. They also report some of the first data on urine levels of a metabolite of this flame retardant.

In recent years, animal studies have suggested that TDCPP is neurotoxic, an endocrine disruptor, and a reproductive toxicant.^4^^,^^5^^,^^6^ The National Research Council has reported TDCPP to be linked to cancer in rats,^7^ and the chemical is on California’s Proposition 65 list of substances known to cause cancer.^8^ However, its potential carcinogencity has not been classified by the Environmental Protection Agency (EPA), the International Agency for Research on Cancer, or the National Toxicology Program.

TDCPP has long been a major flame retardant used for the polyurethane foam padding in furniture and automobiles, according to Joel Tenney, director of advocacy for flame retardant manufacturer ICL Industrial Products. A recent study found it in many U.S. couches,^9^ and it was also the flame retardant found most frequently in foam padding in a survey of 101 products intended for use by infants and young children.^10^ Consumer products are not labeled to reveal which flame retardants they contain.

Scientists analyzing polyurethane foam from products intended for use in homes and offices have documented TDCPP at relatively high levels of up to 5% by weight.^11^ Like other flame retardants used with polyurethane foam, TDCPP is not chemically bonded to the foam, which allows it to escape into indoor environments and accumulate in house dust. The fact that dust can be a source of exposure to flame retardants was first revealed by investigations into why compounds associated with polybrominated diphenyl ether (PBDE) flame retardants were bioaccumulating in people’s bodies. The main PBDE formulation used with polyurethane foam, PentaBDE, was banned in Europe and voluntarily discontinued in the United States after 2004.

The two new studies complement one another to provide a wider picture of current exposure to TDCPP, says Courtney Carignan, the Boston University doctoral student who is first author of one of the studies, published in *Environment International*.^2^ Her team’s research documented the presence of TDCPP’s main metabolite, bis(1,3-dichloro-2-propyl) phosphate (BDCPP), in all of the 24 female and 5 male office workers whose urine was analyzed. The other study, published in this issue of *EHP*, found that 91% of 45 studied men had BDCPP in their urine.^3^

When the compound and its metabolite were detected in the two studies, the concentrations spanned up to four orders of magnitude: The concentrations of TDCPP in dust ranged from < 0.03 µg/g to 326 µg/g, while the urine BDCPP levels were between < 62.1 pg/mL and 25,000 pg/mL.^2^^,^^3^ Unlike PBDEs, which can linger in the body for years, TDCPP is believed to have a half-life of hours or days.

The *Environment International* research showed that the concentrations of TDCPP could be significantly higher in offices and vehicles than in people’s homes.^2^ Belgian researchers have also documented significantly higher levels of TDCPP in dust samples from electronics shops, furniture stores, a pharmacy, and office buildings, compared with homes.^12^

John Meeker, an associate professor of environmental health sciences at the University of Michigan School of Public Health and lead author of the *EHP* study,^3^ says the high levels of TDCPP documented in the new studies may reflect ongoing and direct exposure. He and other experts say the range is so wide because consumers are still bringing the point sources of exposure home from the furniture store or car dealership; plus, cleaning practices and building ventilation can play a role. “The fact that some people have levels that are orders of magnitude higher than others may reflect increased risk to any adverse health effects that may be associated with these chemicals,” Meeker says.

In 2008 the European Union issued a final risk assessment for TDCPP in which it concluded the chemical was safe for its intended uses.^13^ TDCPP was also evaluated by the U.S. EPA’s Furniture Flame Retardancy Partnership, which was convened a decade ago to evaluate the safety of alternatives to PentaBDE.^14^ The literature available at the time linked high-dose exposure to human reproductive and developmental toxicity, carcinogenicity, and genotoxicity. Even so, manufacturers decided to use the compound.

In recent months, however, companies have pledged to discontinue the use of TDCPP, citing the availability of better alternatives. In 2012 ICL Industrial Products announced it would cease production of the compound by 2015 and has already stopped selling it for use in upholstered home items.^15^

In April 2013 both the Consumer Product Safety Commission and UL LLC (formerly Underwriters Laboratory), an independent, nonprofit product safety testing and certification organization, held meetings to address questions about the safety and effectiveness of flame retardants. The UL meeting brought together fire safety scientists, public health scientists, and others “to talk about what we really need for fire safety, review the science regarding safety and exposure to flame retardant chemicals … and set a direction for how we get to a better place,” says Tenney.

But because of the long lifespan of products that contain flame retardants, people’s exposure could continue for years, even decades, despite any industry action. Carignan’s study suggests that regular hand washing may decrease exposure to TDCPP, as it does for PBDEs, and she offers some parental advice to anyone concerned about exposure: “Wash your hands before you eat, and don’t put things in your mouth.”
